# Effects of Caffeine Supplementation on the Recovery of Professional Soccer Players

**DOI:** 10.3390/muscles2010001

**Published:** 2022-12-30

**Authors:** Thais Marques e Silva, Wilson Cesar Abreu, Eduardo Pimenta, Sandro F. da Silva

**Affiliations:** 1 Postgraduate Program in Nutrition and Health, University of Lavras, Lavras 37200-000, Brazil; 2 Studies Research Group in Neuromuscular Responses, University of Lavras, Lavras 37200-000, Brazil; 3 UFMG Soccer Science Center, Sports Department, School of Physical Education, Physiotherapy and Occupational Therapy, Federal University of Minas Gerais, UFMG, Belo Horizonte 31270-901, Brazil

**Keywords:** ergogenic, sports nutrition, performance

## Abstract

(1) Introduction: Soccer players face high demand for training and games. To facilitate their performance, many studies have investigated ergogenic supplements that can assist in the recovery and performance of players. The aim of this research was to assess whether caffeine supplementation can help soccer players’ recovery and performance. (2) Methods: Professional players were given 210 mg of caffeine or placebo in seven games during the state championship, being offered placebo in three matches and caffeine in four matches, administered 30 min before the game, during the game, and after the game. Blood creatine kinase (CK) and heart rate variability (HRV) were measured. Participants rated their perceived recuperation (RPR) and exertion (RPE) on scales developed by Laurent and Borg, respectively. The time that the player spent on the field was also evaluated. *t*-tests and Levene’s test were used to analyze the results. In addition to mean differences, variations in the results were also analyzed. (3) Results: No significant differences were found in CK, HRV, RPR, RPE, or minutes on the field when comparing caffeine supplementation with the placebo. (4) Conclusion: Caffeine supplementation throughout the championship appears to have had no ergogenic effect on athlete performance and recovery.

## 1. Introduction

Caffeine is the subject of much research in football, and it is still unclear about its effect on the recovery of athletes, some studies indicate that caffeine can impair recovery [1], while other research demonstrates that caffeine may have no effect on markers of muscle damage [2] and even some studies suggest that through the antioxidant properties of the substance, it is possible to obtain anti-inflammatory benefits in the muscle after exercise, benefiting the athlete’s recovery [3]. One of the largest issues in this sport is the short recovery time between games; in most soccer championships, players are expected to compete in two games a week [4], as is the case in Brazil. Studies have already established that this interval is shorter than that necessary to achieve complete athlete recovery, increasing the risk of subsequent injuries [5]. In addition, these players are also exposed to major psychological and physical strain due to long trips and other related factors, which can lead to a drop in performance [6].

Nutrition can greatly influence athlete performance in a variety of sports, but nutrition is rarely monitored in professional clubs, as is the case for most soccer training centers in Brazil. Inadequate dietary patterns and the use of supplements without proven efficacy can result in athletes with lower-than-expected physical performance [7].

Therefore, the search for ways to improve athlete performance on the field and recovery in intervals between games has received increasing interest. One method is through supplements and nutrition [8]. Caffeine is one of the most used supplements worldwide due to its beneficial psychological effects and the reduction of fatigue. In the literature, there is no “correct” dose, but it is well established that moderate to high doses of caffeine (3–9 mg/kg) have an ergogenic effect on athletes [9]. However, these doses can cause side effects; the most frequent being nervousness, mental confusion, gastrointestinal disturbance, and insomnia [9]. Caffeine’s possible influence on the recovery of athletes from different sports, including soccer, has recently been studied [10]. At first, several authors proposed that caffeine would impair athlete recovery [11,12] in contrast, other studies assessing muscle damage after caffeine supplementation suggested that despite the increase in sports performance, there are no accompanying differences in muscle damage [13,14].

Considering these findings, it is important to assess whether the use of caffeine promotes an increase in performance without causing increased muscle damage, especially in situations such as official soccer championships, in which there are high-performance demands but congested game schedules, often limiting the ideal recovery time of these athletes. The prescription of this supplement for soccer athletes might provide additional security. Therefore, the objective of this study was to evaluate the effects of caffeine supplementation at moderate doses on the recovery of professional soccer athletes during an official championship.

## 2. Results

### 2.1. Caffeine Consumption

In the first meeting with the players, an interview was held in which they answered questions on general health and on food and supplementation. Caffeine supplementation was not reported by any of the participants, but its consumption as part of the usual diet was observed in 70% of the total sample, and the rest of the players had consumed the substance through supplementation at some point.

Below are the data of the players who consumed caffeine in food, including the amount consumed in household measures and its relative value in milligrams. There was a sample loss of 20%, due to contractual issues of the club.

### 2.2. Relationship between Minutes Played and Caffeine Supplementation

**Figure 1:**
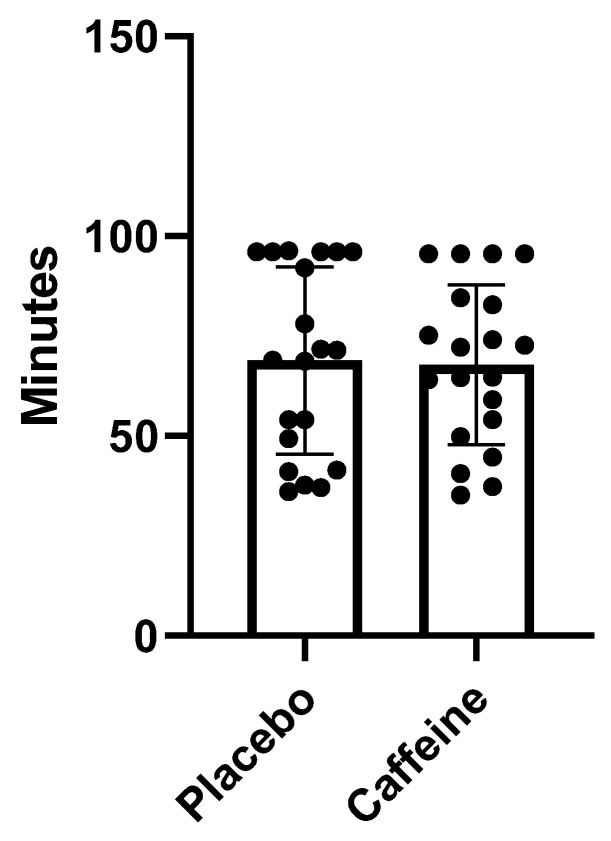
Minutes played: Caffeine vs. Placebo.

There was no significant difference (*p* > 0.88) between caffeine and the placebo groups in terms of minutes played. A reduction of 9.71% was observed when athletes consumed caffeine (Figure 1).

### 2.3. Relationship between Rating of Perceived Exertion (RPE) and Caffeine Supplementation

**Figure 2:**
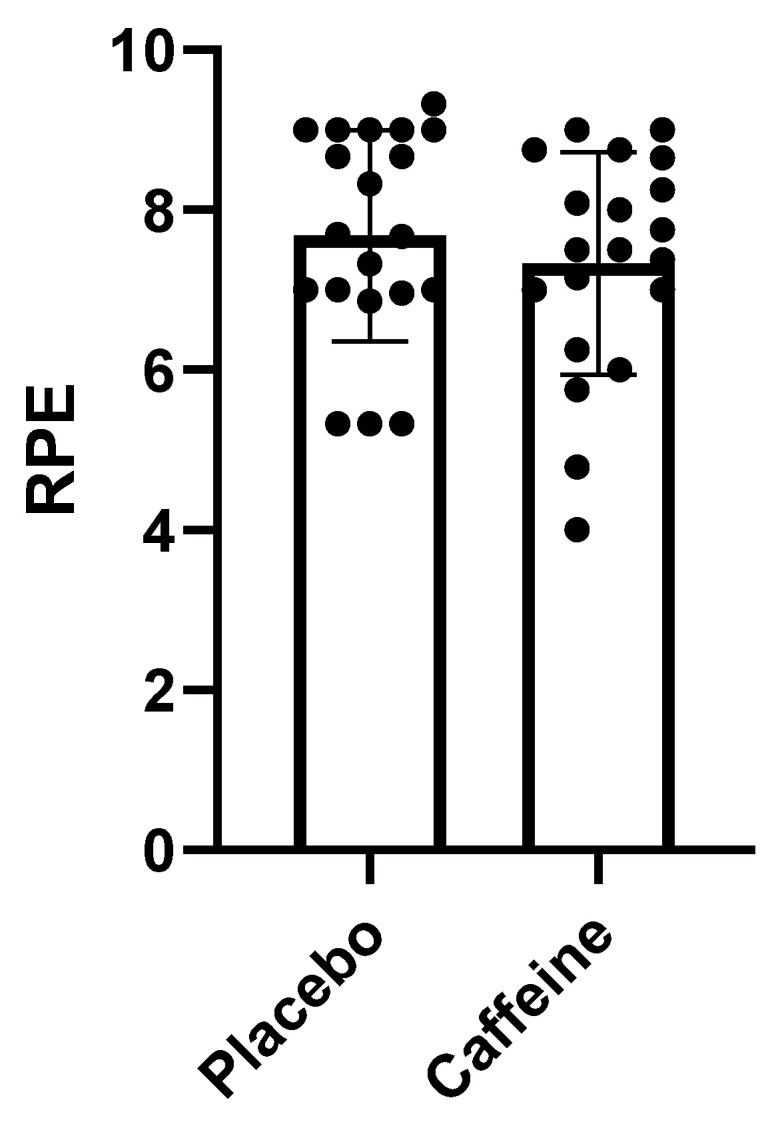
RPE: Caffeine vs Placebo.

A 3.72% reduction in the RPE of athletes was observed when caffeine was offered (Figure 2), but there was no significant difference (*p* > 0.42) between caffeine and the placebo. Below is a graph showing the RPE of individuals that consumed caffeine and the placebo.

### 2.4. Relationship between Rating of Perceived Recovery (RPR) and Caffeine Supplementation

**Figure 3:**
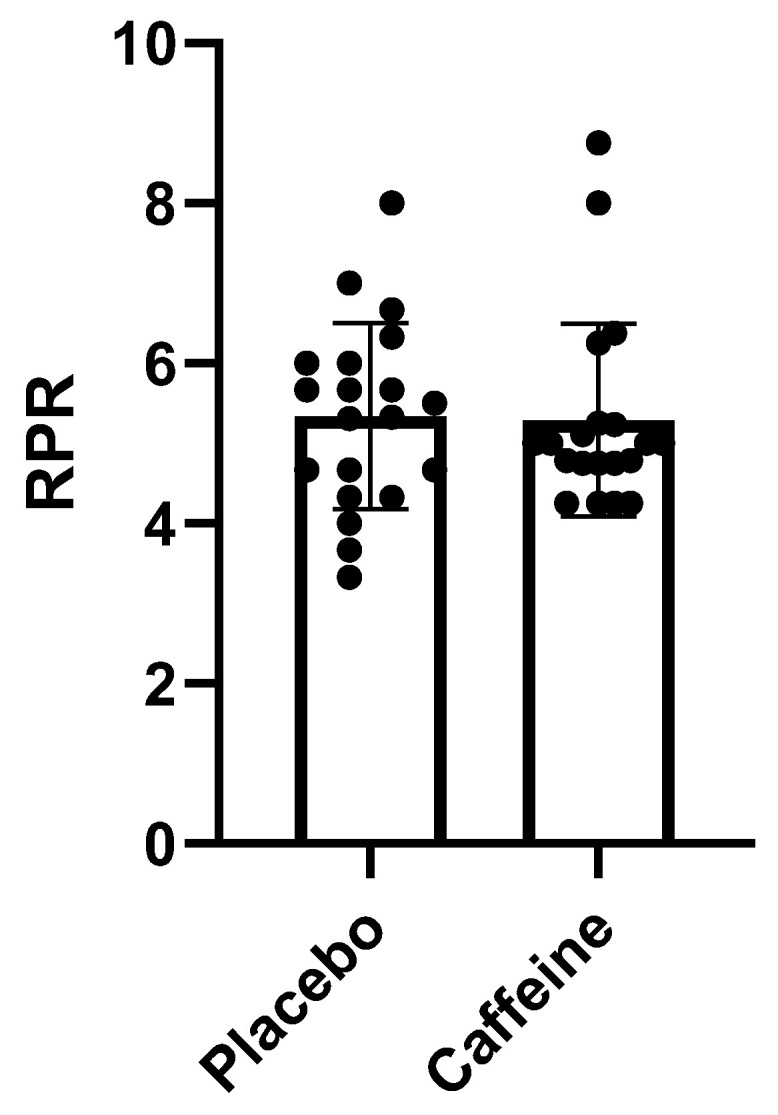
RPR: Caffeine vs. Placebo.

Regarding the athletes’ recovery, a 1.43% improvement in the athletes’ perception of recovery was obtained, but this was not statistically significant (*p* > 0.89). The graph below shows the individual recovery outcomes comparing caffeine and placebo consumption (Figure 3).

### 2.5. Relationship between Basal Creatine Kinase (CK) and Caffeine Supplementation and Placebo

**Figure 4:**
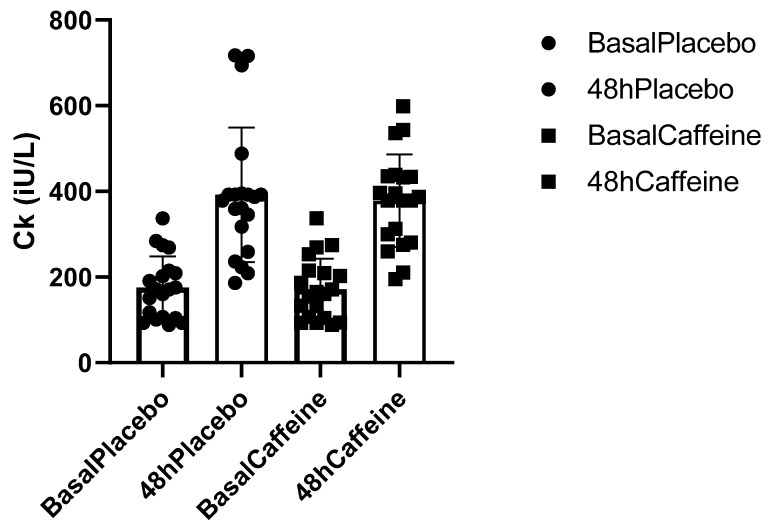
CK: Caffeine vs. Placebo.

Basal CK was measured before the beginning of the Minas Gerais championship and served as a parameter for comparison (Figure 4). When we compared the basal CK with the athletes’ CK elevation during caffeine and placebo supplementation, we found that the basal CK was 3.28% lower (indicating less inflammation), but this difference was not statistically significant (*p* > 0.87). When the comparison was between the values of basal CK and CK 48 h after caffeine supplementation, plasma CK was 5.08% lower after caffeine supplementation. In the graph below, it is possible to observe individual differences in CK 48 h after placebo and caffeine supplementation.

### 2.6. Relationship between the Recovery Test and Caffeine Supplementation

**Figure 5:**
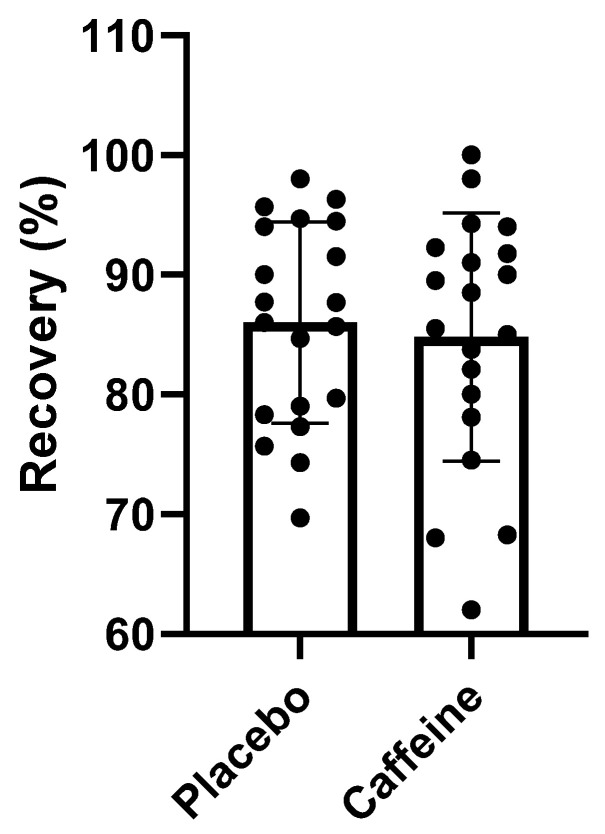
Recovery Test: Caffeine vs. Placebo.

A 3.20% improvement in Heart rate variability (HRV) was obtained when caffeine was offered compared to a placebo (Figure 5), but this difference was not statistically significant (*p* > 0.69).

## 3. Discussion

In this study, caffeine supplementation for professional soccer players during a championship did not seem to provide ergogenic effects. These findings can be explained by several factors, such as genetics and the psychological effects of the championship. There was no improvement in muscle damage, perception of recovery, and perception of exertion in these athletes.

### 3.1. Caffeine and Individuality

Participants who reported dietary caffeine consumption reported daily consumption, with a mean intake of 183,085 (±32.484) mg/day. The average amount of caffeine in 100 mL of brewed coffee is standardized at 71.2 mg [15] Individuals who consume caffeine usually tend to need higher doses to obtain the same effects as those obtained by individuals who do not consume caffeine. Physiological habituation to caffeine can occur within 20 days of continuous consumption [16].

Another important fact about caffeine consumption, metabolism and its consequent effects on an athlete’s body is genetic variation. In recent years, genes that control differences in the metabolic rate of individuals have been studied, resulting in the categorization of slow and fast metabolizers, depending on the CYP1A2 gene. Fast metabolizers seem to receive caffeine’s performance-enhancing effects without major side effects, whereas slow metabolizers may not experience any effect of caffeine with consumption or even a decrease in performance with caffeine supplementation. High consumption of caffeine by slow metabolizers also increases the risk of important side effects, such as hypertension and cardiovascular diseases [17].

Athletes were deprived of dietary consumption of caffeine on match days to avoid excessive doses of caffeine or the interference of caffeine consumption with the results found during collection. During an athletes’ concentration period, the hotels were provided with the menus to be offered ahead of time, both to avoid providing athletes with caffeinated substances, and to standardize the meals that preceded the measurements, thus avoiding the influence of the consumption of other foods or substances on the results obtained.

Considering the individual differences in caffeine consumption and tolerance reported by the athletes, we conducted two tolerance tests to select a safe dose for the athletes. We initially offered a dose of 210 mg and as it was well accepted, we increased the dose to 420 mg. However, at this dosage, one of the athletes reported anxiety and difficulty sleeping after the end of the game, so the lower of the two dosages was selected as it was sufficient to produce ergogenic effects without producing side effects [9].

### 3.2. Influence of Caffeine on Minutes on the Field

There was no significant effect of caffeine supplementation on minutes in the field or RPE, demonstrating that athletes’ performance did not increase during the match due to the substance. Studies on caffeine in soccer are not often performed with players in an official match due to the difficulty of intervening in this period. The results obtained by this study during the official championship diverge from those found in specific tests or simulated matches [18,19,20]. A possible explanation for this fact is that the circumstances of an official match greatly influence the athlete’s psychological state and consequent performance. Since caffeine is a psychoactive substance, its effects can be greatly modified when comparing simulated matches or specific tests to games at home or away from home, among others [21]. An article by Metaxas [22] observes that external factors, such as the quality of the opponent and the degree of motivation of athletes, can also alter game performance.

Another important factor that differentiates official matches from simulated tests is the match start time. Athletes have been found to perform differently in matches according to their endogenous rhythms and match times [23,24].

Knowing that match start times are alternated, we alternated between caffeine and placebo supplementation for the morning, afternoon and night matches so that the experiment was not systematically influenced and still yielded legitimate results, which is not a concern for most studies that conduct simulations, so it can explain the divergence in the results. It is important to remember that in official matches, time on the field can also be influenced by factors such as substitutions due to injuries, sending-offs and game strategies.

### 3.3. Influence of Caffeine on Athletes’ Recovery

Monitoring athlete recovery is of great importance for making necessary individual adjustments, minimizing injuries, improving well-being and restoring physical performance, which is why recovery markers greatly assist prescription [25].

In the present study, none of the recovery markers (RPR, HRV, and CK) showed significant differences with or without caffeine supplementation. With caffeine supplementation, fatigue is expected to be delayed, and this can influence recovery parameters; delayed fatigue induces an increase in workload, which can generate an excessive increase in physical stress, thus generating doubt regarding its beneficial effects on athlete recovery [13]. Another factor that may explain the lack of significant differences in recovery parameters is the fact that some athletes have been reported to experience sleep deprivation after caffeine supplementation, especially in night games, thus impairing the athlete’s recovery [12]. Accordingly, some studies even found that caffeine consumption was harmful to athlete recovery [26]. Other studies that evaluated the effect of caffeine on RPE also failed to find beneficial effects of caffeine supplementation on the perception of exertion, similar to what was found in this research [14,27].

CK is a biomarker that can help to prevent overtraining and to assess the efficiency of athletes’ recovery [28]. In the present study, supplementation with caffeine did not promote significant changes in CK concentrations compared to the administration of the placebo. Most studies that have investigated the effects of caffeine supplementation on CK also did not find significant differences [29,30]. CK, when evaluated individually, may not be a good recovery marker; it has major interindividual alterations, and likely should always be considered with other markers. Another factor that must be considered is the collection time. A study conducted by Silva [25] demonstrated that 72 h postgame was still insufficient time to restore homeostatic balance, especially when analyzing CK.

## 4. Materials and Methods

### 4.1. Sample

The sample consisted of 19 professional soccer players who were part of a soccer club in Minas Gerais, Brazil. This involved convenience sampling; that is, the individuals recruited for this survey were selected because they were readily available. The study was approved by the Ethics Committee for Research with Human Beings, and its Certificate of Presentation for Ethical Appreciation number was 20221419.7.0000.5148.

All participants were at the first division competition level and had a history in the sport of at least 5 years as a professional, training approximately 3 h each day. The mean age of the sample was 24 ± 4.20 years, the average height was 1.80 ± 0.079 m, the body mass was 79.28 ± 8.92 kg, the body fat was 9.75 ± 0.08% g, the peak heart rate (HRpeak) was 195.08 ± 7.55 bpm and the maximum oxygen volume (VO2_MAX_) was 52.34 ± 3.50 mL·kg·min^−1^. None of the participants were smokers or currently on other medications. Players did not supplement their caffeine dose with other stimulants during the research period. On the other hand, 48% of the participants incorporated caffeine in their daily diet at approximately 183,085 (±32,484) mg/day, equivalent to a cup or two cups of coffee [15]. All club athletes who were active for a substantial amount of time during matches throughout the championship were included in the survey.

### 4.2. Experimental Design

**Figure 6:**
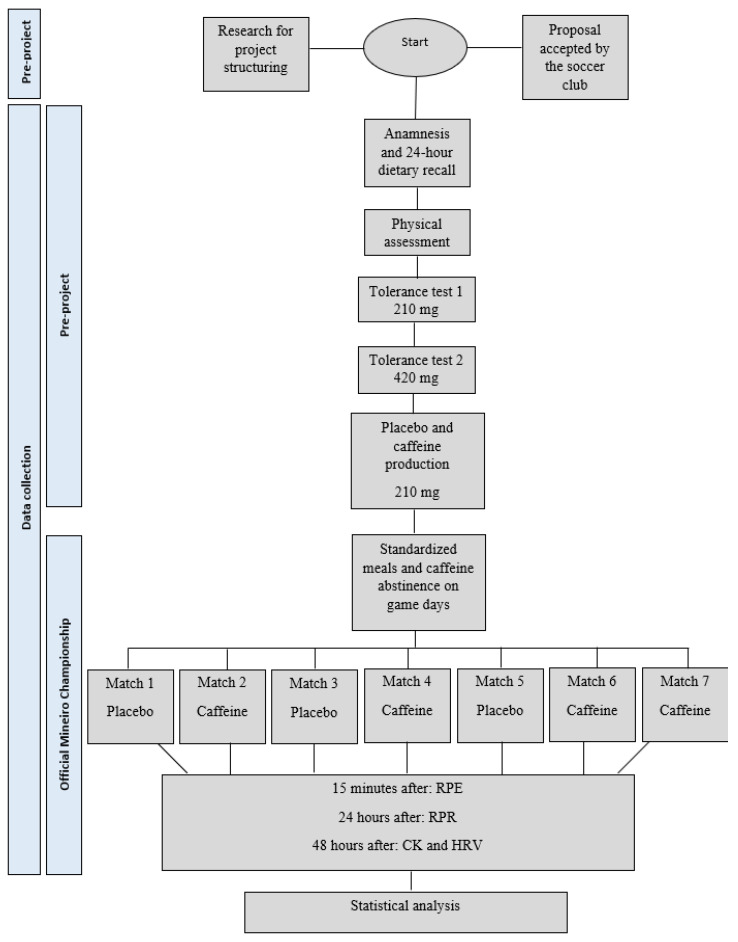
A flowchart containing the stages of the research.

Figure 6 is a flowchart containing the stages of the research. The study was a single-blind, placebo-controlled intervention that was conducted from October 2019 to March 2020 and covered the preseason period and the Official Mineiro Championship.

#### 4.2.1. Physical and Nutritional Assessment

At the initial meeting at the football club, the players individually answered an anamnesis with questions about health, sports history and supplementation and eating habits. At this first meeting, a physical evaluation was also conducted. The athletes were weighed on a digital platform scale, and height measurements were performed using a fixed stadiometer. At this time, physical evaluations were conducted to define the body fat percentage, using the Pollock protocol of seven skinfolds made through the scientific adipometer.

##### Physical Assessment

In the first meeting, the first anthropometric assessment of the athletes was conducted. Athletes were weighed on a platform scale and their height was measured with a fixed stadiometer and body fat percentage was assessed using a scientific adipometer for which Pollock’s (1984) protocol of seven skinfolds was used, namely, pectoral, subscapular, suprailiac, middle axillary, triceps, abdominal and thigh, with measurements always being taken from the athlete’s right side. The evaluation was always conducted by the same evaluator, always in the morning, before the start of training.

##### Nutritional Assessment

Initially, an individual nutritional consultation was conducted, in which an anamnesis containing questions about health, eating habits and supplementation was applied. A 24 h dietary recall, which is frequently used to assess the nutrition of athletes, was also collected, along with the athlete’s sports history. The dietary recall was always held on Wednesdays to avoid interference from days off or travel since the participating athletes lived in a hotel that provided them with standardized meals.

After this initial consultation, individual menus were calculated following the nutritional recommendations for soccer athletes and containing points that should be addressed for the purposes of clarity and increased adherence. For calculating energy expenditure and dietary prescription, values for carbohydrates between 7 and 10 g/kg of body weight were used; for proteins, values between 1.4 and 2.0 g/kg of body weight were used and for lipids, values from 0.4 to 1 g/kg of body weight were used—always considering the athlete’s position and their individual requirements previously established during the consultation. To calculate the meal plans, Webdiet^®^ 2.0 software was used. Appropriate changes were made to the menus of the hotel where the athletes lived so that it was easier for athletes to adhere to the food plan. After these changes, a new meeting was held with the athletes and the technical committee, to emphasize the importance of adherence and answer questions regarding food planning.

The calculations of energy expenditure of athletes were made using the recommendations of the National Research Council [16] if the physical activity of athletes ranged from intense to very intense, which is from 50 to 59 kcal/kg of body weight/day. During the preseason, individuals’ physical activity was considered very intense due to the greater requirements of training; during the season, physical activity was reduced to intense, along with a small accompanying reduction in caloric intake. Throughout the experiment, nutrition was monitored and necessary changes were made according to the requirements of the athletes on an individual basis.

#### 4.2.2. Caffeine Tolerance Tests

Two caffeine tolerance tests were conducted during friendly matches in the first two friendly games. Players ingested 210 mg in the first game and 420 mg in the second game to determine the safest dose to offer. After the game in which 420 mg of caffeine was provided, one of the players had adverse reactions such as anxiety and insomnia, so the safe dose was established at 210 mg.

After establishing the dosage, placebo capsules identical to those containing the caffeine were made, each containing 210 mg of maltodextrin.

The testing period commenced at the start of the 2020 Minas Gerais Championship, between 22 January and 1 March, when players began caffeine or placebo supplementation. The hotels where the athletes stayed had received a menu ahead of time with the food and quantities that should be offered during the stay, including the pregame meal, thus standardizing the athletes’ meals. Athletes were requested to abstain from caffeine on competition days. Caffeine supplementation in the form of capsules was provided 30 min before games, during the warm-up period, along with 100 mL of water to aid ingestion. Placebo was offered in three matches and caffeine in four matches.

#### 4.2.3. Performance Markers

##### Rating of Perceived Exertion (RPE)

The Borg scale rating of perceived exertion (RPE) (1982) was used after the end of the games. Approximately 15 min after the games finished, players rated on a scale of 0 to 10 how much they felt they had tired in that game. This protocol was followed for the seven games, for all athletes participating in the research.

##### Rating of Perceived Recuperation (RPR)

Approximately 24 h after the game, the players answered the Laurent scale of the rating of perceived recuperation (RPR) [17], where they individually rated on a scale from 1 to 10 how much they perceived they had recovered between games. Athletes were already familiar with this scale, as it is frequently used by the club.

##### Creatine Kinase (CK)

Forty-eight hours after the end of the game, 32 μL of blood was collected from each athlete through perforation with an Accu-Check Softclix^®^ lancing device and placed on the strip so that blood CK levels could be analyzed using the biochemical analyzer Reflotron Plus^®^.

##### Recovery Test (Heart Rate Variability—HRV)

After blood collection, the players went to take the recovery test, which is based on the athletes’ heart rate variation. This test was performed using the FirstBeat TeamBelt^®^ device, which is a chest strap. After having the brace placed on their chest, the athletes lay down and rested, and then the analysis was performed for 3 min. This test was performed on all players simultaneously.

### 4.3. Statistical Analysis

Descriptive statistics were reported, including the mean, standard deviation, and standard error of the mean. The collected data were analyzed by statistical tests. The Shapiro–Wilk normality test was used to identify the distribution of parametric data. For the analysis of the variables, an independent-samples *t*-test was used to assess the equality of means and the Levene test was used to assess the equality of variances. A difference was considered significant when *p* < 0.05. In addition to comparing the means, the variation (Δ) of the results was calculated. Statistical calculations were performed using SPSS^®^ version 25.0 statistical software, and graphs were plotted using Prism^®^ version 8.0 software.

## 5. Conclusions

Another point that may have limited the effect of caffeine is caffeine tolerance by athletes who already use it continuously, and therefore, do not tend to experience effects when exposed to the substance.

Caffeine supplementation seemed to have no ergogenic effect on the performance and recovery of professional soccer athletes during an official championship, but further studies are needed.

Another assessment that could have added value to the research is the distance covered by players in the field, which would benefit future research.

## Data Availability

The datasets used and/or analysed during the current study are available from the corresponding author on reasonable request.
